# Modern Methods for Postural Correction: The Impact of Kinesio Taping and Corrective Exercises on Dentists—A Quasi‐Experimental Study

**DOI:** 10.1002/hsr2.71242

**Published:** 2025-09-12

**Authors:** Zahra Fadaei Forghan, Parisa Sedaghati

**Affiliations:** ^1^ Department of Sports Injuries and Corrective Exercises, Faculty of Physical Education and Sport Sciences University of Guilan Rasht Iran

**Keywords:** kyphosis, physical activity, posture, tape

## Abstract

**Background and Aims:**

This study aimed to investigate the effectiveness of a combination of comprehensive corrective exercises and Kifnesio taping on postural abnormalities in dentists with forward head postures, as a group with a high risk of musculoskeletal disorders caused by incorrect working postures.

**Methods:**

This quasi‐experimental study employed a pre‐test–posttest control group design. The participants were dentists from Rasht, Iran, with more than 5 years of professional experience. Thirty dentists with forward head posture (FHP) (angle > 46°) volunteered to participate in this study and were randomly divided into two experimental and control groups (*n* = 15 per group). The experimental group performed corrective exercises with the KT method for 8 weeks (three sessions per week). The comprehensive corrective exercise protocol included integrated release and strengthening exercises aimed at postural muscles. KT was applied in a U‐shaped taping on the upper trapezius and cervical spine. The control group did not receive any intervention. In the pre‐test and posttest stages, the evaluated variables were FHP and kyphosis angle. Data were analyzed using repeated measures ANOVA at a confidence level of 99%, with a statistical significance of (*p* < 0.01) by SPSS V25.

**Results:**

The results indicated significant differences in the dentists' FHP (*p* = 0.001, effect Size = 0.61), rounded shoulder (*p* = 0.007, effect size = 0.23), kyphosis angle (*p* = 0.001, effect size = 0.61), and scapular position 0° (*p* = 0.001, effect size = 0.44), 45° (*p* = 0.001, effect size = 0.36), 90° (*p* = 0.001, effect size = 0.32).

**Conclusions:**

In conclusion, dentists' postural alignment significantly improved when a comprehensive corrective exercise was implemented together with concurrent KT of the thoracic and scapular regions. Specifically, there were clear improvements in thoracic kyphosis, forward head posture, rounded shoulders, and scapular alignment, indicating that the intervention was successful in correcting typical musculoskeletal abnormalities in this population.

**Trial registration**: IR.SSRI.REC.2021.1208; dated: 24.08.2021.

## Summary

- This study introduces a novel approach by demonstrating that the combination of corrective exercises and kinesiology taping (KT) effectively improves postural alignment in dentists.
- Our comprehensive intervention protocol resulted in improvements in scapular positioning, forward head posture (FHP), rounded shoulders, and thoracic kyphosis.
- To the best of our knowledge, this is the first study to evaluate the effectiveness of KT combined with corrective exercises specifically for addressing FHP in dentists.

## 1 Introduction

Musculoskeletal disorders (MSDs) are one of the most common and significant challenges in the dental profession. Findings have shown that the prevalence of neck musculoskeletal pain has been reported to range from 19.8% to 85%. Studies have identified the most important factors contributing to the risk of MSDs as sustained poor postures during work and incorrect work habits [1]. A good posture allows the muscles of the body to function with maximum efficiency. In addition, in a good standing posture, the body's joints are in a state of equilibrium (vertical and rotational forces are balanced) with the least amount of physical energy being used to maintain this upright position [2]. On the other hand, musculoskeletal disorders are described as disorders of the muscles, nerves, tendons, ligaments, joints, cartilage, or vertebral discs [3]. According to a previous study, more than 90% of dentists' postures during work were associated with moderate to high‐risk levels of MSDs and in some dental procedures, such as surgeries, were the least favorable [4, 5]. A study on body posture reported that dentists spent 86% of their working time with a bent neck of at least 30° and 52% of their time with a bent trunk posture of at least 30° [6]. The main reason for these poor body postures is to achieve an ideal view of the patient's mouth and to provide patient comfort [7]. In another study conducted in India, the most common cause of dentists' pain was being seated for a long time while working, with a prevalence of 63.3% [8]. Additionally, in a study by Chiou et al. on the musculoskeletal disorders of dentists, the results showed that 48% of dentists had neck injuries and 37% had shoulder injuries [9]. Moreover, the results of a study on 150 dentists in Tehran Province, Iran, showed the highest prevalence of musculoskeletal disorders in the neck and shoulders (50%, 45.3% respectively) [10].

In this regard, exercise therapy, manual therapy and physical therapy are used as complementary methods for the treatment of musculoskeletal disorders. It has been recommended in various studies to correct the posture of the cervical spine and trunk [11, 12]. Since researchers are seeking ways to make the training sessions shorter and more effective [13, 14], there is ongoing research on effective training protocols. The Kinesio taping (KT) method is another intervention used for patients with musculoskeletal disorders. Generally, there are six basic concepts in the corrective KT method, consisting mechanical, fascial, spatial, ligamental, functional, and lymphatic items [15]. Two common taping methods are: U‐shaped and I‐shaped taping [16]. Recent research has explored the use of kinesio taping (KT) as a novel therapeutic intervention [17, 18], with its proposed mechanisms including mechanical support to soft tissues, enhancement of local blood and lymphatic circulation, prevention of excessive muscle strain, and improvement of proprioceptive function [15, 19, 20, 21]. Studies have shown that the U‐shaped method may be more effective than the I‐shaped method in improving proprioception and increasing neuromuscular retraining, especially in conditions such as forward head deformity and shoulder hunchback. This is because its multidirectional coverage involves more areas of skin and sensory receptors, which provides more continuous mechanical and sensory feedback. Therefore, the U‐shaped method has a relative advantage over the I‐shaped method in conditions where the goal is to correct complex movement patterns or combined postural deformities [22].

The advantage of KT, as a noninvasive modality, is increasing the body′s natural capacity for self‐repairing. In addition, KT improves distress, diminishes inflammation, alters locomotor activity in the muscles, stimulates proprioception, helps lymphatic drainage and blood circulation, and accelerates tissue growth [23]. In this regard, Sheng Shih compared the effects of KT and therapeutic exercises for 5 weeks on forward head posture in people. The findings showed that both taping methods and corrective exercises were effective in improving body posture indices, cervical spine range of motion, and neck function compared to the control group. However, therapeutic exercises had a greater effect on improving the angle of the lower cervical vertebrae and the range of motion of lateral bending than taping. As far as current evidence suggests, scapulothoracic exercises can effectively improve joint position sense and postural stability in these individuals [24]. Accordingly, it can be concluded that although both approaches are useful in improving forward head posture, corrective exercises are more effective in improving musculoskeletal function [23]. However, few studies have been conducted to investigate the effect of the KT to improve posture [18, 25].

Dentists are known as one of the occupational groups with a high risk of developing musculoskeletal disorders, especially postural abnormalities [26]. The professional activity conditions of this group require maintaining static, bent positions in the neck and trunk for a long time, which leads to an increase in asymmetric mechanical load on the cervical and dorsal spine as well as the muscles of the shoulder area. These factors cause the creation of compensatory movement patterns, reduced muscle balance, limitations in the range of motion and ultimately the occurrence of abnormalities such as forward head posture (FHP), rounded shoulders and hyper kyphosis [4, 26]. So, there is a growing need for comprehensive preventive and therapeutic interventions. Strategies such as corrective exercises, correct posture training, the use of ergonomic equipment, and complementary techniques such as taping therapy or yoga can play an important role in reducing the physical burden of the job [27]. By reviewing articles on the occupational musculoskeletal disorders of dentists, it was found that only a few studies have investigated the effects of modern movement‐based therapy methods on the elimination of occupational musculoskeletal disorders [28, 29, 30]. Also, researchers are using different approaches and combinations of corrective exercises with other modalities to improve the effectiveness of treatment and facilitate early correction of postural disorders [31]. Comprehensive corrective exercises that include soft tissue and muscle release, strengthening of weakened muscles, and motor integration to retrain correct movement patterns can be effective. These exercises are designed to restore muscle balance, improve postural stability, and reduce the load on spinal structures [32, 33]. Along with corrective exercises, KT, as a complementary and noninvasive method, helps improve postural awareness, reduce muscle tension, and correct neck and shoulder posture during daily activities by providing continuous stimulation of proprioceptors [17, 18]. Taping can transmit corrective nerve signals to the central nervous system by limiting undesirable ranges of motion and amplifying the effects of corrective exercises throughout the day. Despite the evidence on the separate effects of corrective exercises and KT, few studies have examined the effectiveness of a combined intervention of these two approaches in high‐risk populations, such as dentists [26]. The present study was designed and implemented in response to this study gap and to investigate the synergistic effects of these two interventions on postural indices, functional mobility, and musculoskeletal performance indices in dentists. Therefore, in the present study, we investigated the effect of an 8‐week combined intervention consisting of KT (aimed at preventing excessive muscle tension during dental procedures) along with corrective exercises on postural alignment, including forward head posture, kyphosis angle, and rounded shoulders, in dentists with forward head posture (FHP).

## 2 Methods

### 2.1 Study Design and Participants

This study is a quasi‐experimental and applied research with a pre‐test and post‐test design, conducted under controlled and uniform conditions for participants. All participants received general information about the experimental design, potential risks, and benefits of the study and signed the informed consent form before participating in the study. Also, all experiments were conducted according to the latest version of the Declaration of Helsinki.

The study population consisted of male and female dentists (35–50 years old) who had more than 5 years of work experience in Rasht, Iran. In the initial review of 38 dentists, 30 dentists with more than 5 years of work experience and FHP with an angle greater than 46° were included in the research based on the inclusion and exclusion criteria.

**Figure 1:**
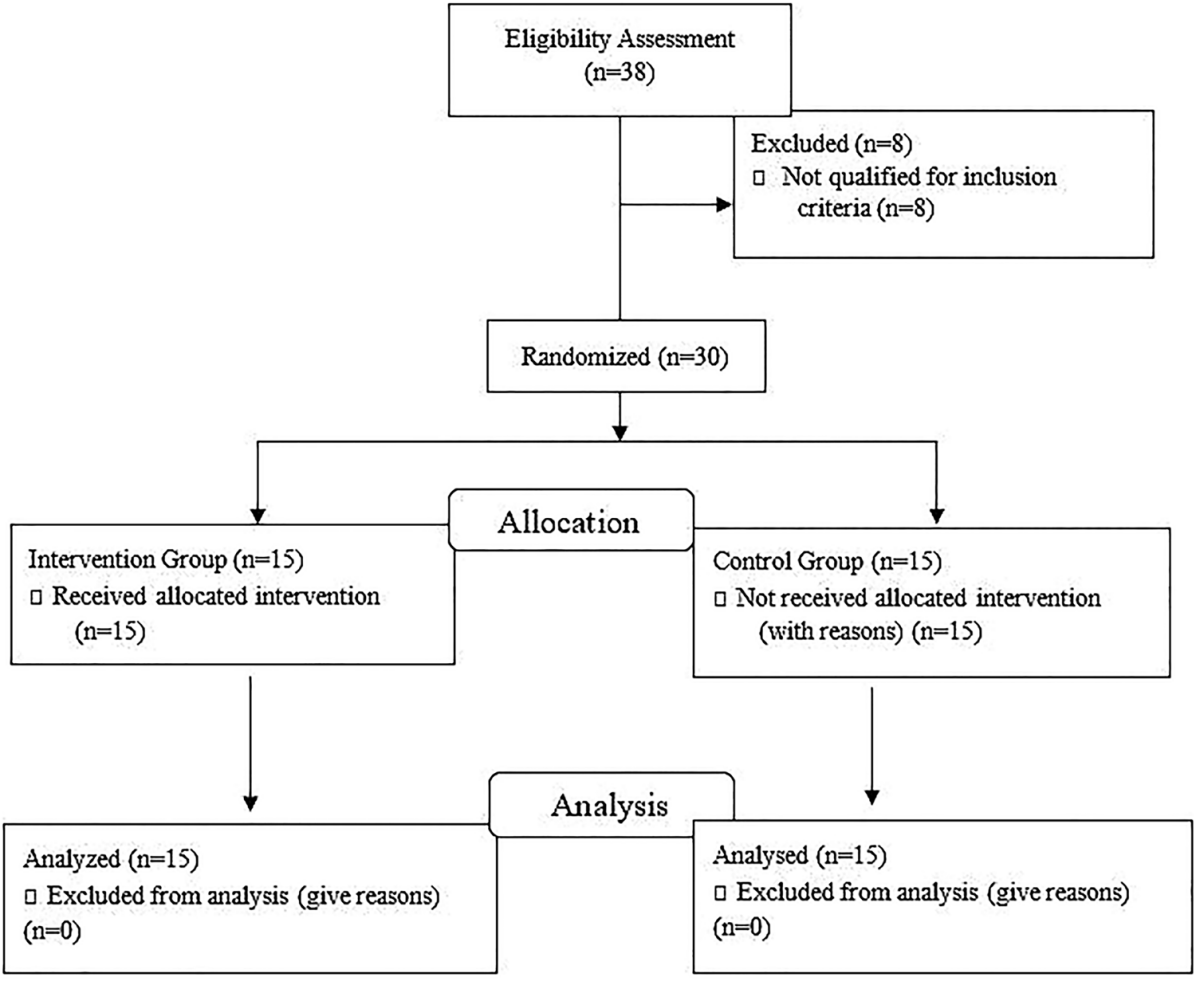
Participants flow diagram.

The sample size for the current study was calculated based on a previous study [30] using G*Power software, Given a *p* value of 0.05, a power of 80%, the forward head posture (FHP) angle, and a reasonable effect size (0.5) for the minimal clinically significant difference, 30 dentists for the whole experiment were selected for this study via purposive sampling according to the inclusion criteria. Fifteen volunteers would be required for each group (Figure 1). This study randomly divided participants into two groups through simple randomization. To reduce selection bias, allocation concealment was prepared by assigning numbers to participants on the list. Using a simple random allocation approach, odd‐numbered participants were assigned to the experimental group and even‐numbered participants to the control group. The allocation list was generated by a researcher not involved in outcome assessments.

This resulted in the formation of the corrective exercise group (CEG), consisting of 15 participants, and the control group (CG), which also comprised 15 participants. The two groups were matched in terms of years of work and sex, with each group consisting of six males and nine females. The study population consisted of dentists with more than 5 years of work experience, who had FHP with an angle greater than 46° [34, 35, 36] and worked in Rasht clinics (Rasht, Iran). Participants (age = 42.34 ± 5/63 years, body mass = 69/20 ± 11/59 kg, height = 167 ± 9.71 cm and body mass index (BMI) = 24.69 ± 3/73). In an 8‐week combined intervention, the experimental group performed the selected corrective exercises, along with the KT method 3 days a week, whereas the control group continued their usual daily activities.

### 2.2 Inclusion Criteria

The inclusion criteria were as follows: dentists, male and female, with more than 5 years of work experience who had FHP with an angle ranging from 46 to 55 degrees, no severe kyphosis greater than 58°, willingness to participate voluntarily in the study.

### 2.3 Exclusion Criteria

The exclusion criteria were as follows: participants with structural spinal deformities such as scoliosis, hyper kyphosis exceeding 60°, history of cervical spine surgery, or neurologic or vestibular disorders a history in the past year of fracture, joint diseases, neck pain or radiculopathy and absence of more than 2 days of training [37, 38].

### 2.4 Testing Procedure

*Postural assessment*. FHP angle is most often described as excessive anterior positioning of the head in relation to a vertical reference line [39]. The FHP angle was measured using side view photography, the angle of a vertical line passing through the spinous process of the seventh cervical vertebra (C7) and the line joining the tragus of the ear [40] and was analyzed using the kinovea software [41]. The FHP angle increase indicates development, the validity of this method was estimated at 0.95 [42]. Next, to assess the kyphosis angle, T2 and T12 landmarks were identified and marked. Using a flexible ruler, the curvature of the spine was plotted on an A3 paper, and an arc was drawn between the two landmarks [40]. The two ends of each curve were connected with a line, called “L”, and a line perpendicular to L was drawn from the peak of the curve, called “H”. The curvature angle was calculated using the following formula: *Ɵ* = 4 Arctan (2 h/L) [43, 44]. The ICC test of flexible ruler (Intra‐tester reliability) at 0.92 and the validity of this method was estimated at 0.90 [43]. Additionally, to assess the rounded shoulder posture, the participant was asked to stand next to a wall to mark the acromion process, and was measured by a double square ruler [45, 46]. The distance between the shoulder and the wall was then calculated on both sides (right and left) and averaged. Kibler confirmed the validity and reliability of the double square ruler. The intraclass correlation coefficients for intra rater reliability ranged from 0.89 to 0.9 l [45, 46].

**Figure 2:**
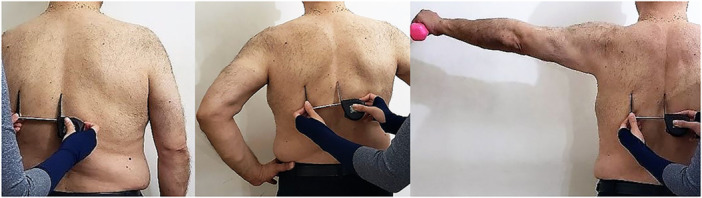
Kibler test angle 0°–45°–90°.

*Kibler′s lateral scapular slide test:* Kibler's lateral scapular slide test was used in this study to evaluate the scapular position. This test was used to evaluate scapular asymmetry and to determine the ability of scapular stabilizer muscles at 0°, 45°, and 90° of shoulder abduction (Figure 2). In this test, first, the lower angle of the scapula was marked with a marker, and then, its distance from the adjacent vertebra was measured in three positions (hands held next to the body at 0°, hands placed on the head of the femur, and hands in 90° abduction with maximum internal rotation while holding a 1‐kg weight in hands). If there was a difference of 1.5 cm or more between the two scapulae, the test result was considered positive. Kibler estimated the intragroup reliability of this test at 0.84–0.88, and between groups, reliability was estimated at 0.77–0.84 at different angles [47].

*Anthropometric measurements:* Height was measured to the nearest 0.1 cm and weight was measured to the nearest 0.1 kg with the use of a scale and length meter (Seca, IN, Japan). To increase the reliability of the tests, all assessments were conducted by the researcher.

## 3 Intervention

The intervention consisted of two parts. The practicability of the experimental group were determined in a pilot study with full details of volume, intensity, and type of exercises; National Academy of Sports Medicine (NASM) includes deep neck flexor strengthening, pectoralis major stretching, scapular stabilizer strengthening (Table 1) along with U‐shaped taping method on the upper trapezius and cervical spine. Participants assigned to this experimental group will participate a 3‐week NASM exercises along with the KT method for 1‐h duration of each session. All subjects were advised to adopt a correct posture during daily activities and exercise in the research. The control group did not receive any intervention.

**Table 1:** A brief description of corrective exercises and the duration or frequency of them.

No	Type, aim of exercise	Type of exercises	Duration (s) or frequency of the exercises	Tools used for exercise progression
1	Releasing the muscles of sternocleidomastoid	Mobility/stretching	30–90 (s), 1 set	A small massage ball
2	Releasing the upper trapezius muscle, levator scapular muscle	Mobility/stretching	30–90 (s), 1 Set	A small massage ball
3	Stretching of the sternocleidomastoid, upper trapezius and levator scapular muscles	Mobility/stretching	30 (s), 1–4 set	—
4	Stretching of the pectoralis major and minor muscles on the side of the wall	Mobility/stretching	30 (s), 1–4 set	Body weight
5	Stretching the shoulder muscles on the side of the wall	Mobility/stretching	30 (s), 1–4 set	Body weight
6	Chin tuck	Strengthening/mobility	10–15 rep, 1–2 set	± TheraBand
7	A floor‐prone cobra	Strengthening/mobility	10–15 rep, 1–2 set	
8	Scaption in prone position/scaption on Swiss ball	Strengthening/mobility	10–15 rep, 1–2 set	± Dumbbells/Swiss ball
9	Retraction of the scapula with traband along with the chin‐tuck movement	Strengthening/mobility	10–15 rep, 1–2 set	± TheraBand
10	Squat with Swiss ball and overhead press with dumbbells	Strengthening/mobility	10–15 rep, 2–3 set	± Dumbbells/Swiss ball

### 3.1 The Protocol of Corrective Exercises

The first part comprised a set of corrective exercises designed by the National Academy of Sports Medicine (NASM), including corrective exercises to adjust the posture of the neck and thoracic spine. This set of exercises, including inhibition, lengthening, activation, and integration techniques, was designed in a systematic process to meet all the training goals and to eliminate neuromuscular disorders [32]. Each session included warm‐up exercises for 5–10 min, corrective exercises for 20–40 min, and cooling down for 5–10 min.

A total of 10 exercises were performed in the exercise sessions. The first exercise involved releasing the neck muscles (sternocleidomastoid muscle) using a small massage ball. The second exercise involved releasing the upper trapezius muscle and levator scapulae with a small massage ball. The third exercise included stretching the sternocleidomastoid, levator scapulae, and upper trapezius muscles. The fourth exercise involved stretching the large and small pectoral muscles next to a wall. The fifth exercise involved shoulder stretching next to a wall. The sixth exercise included chin tucks (with and without a TheraBand). The seventh exercise consisted of cobra movements on the ground. The eighth exercise consisted of a prone scaption with and without dumbbells and a scaption with dumbbells and a ball. The ninth exercise consisted of scapular retraction with a band, along with chin tucks, and finally, the tenth exercise consisted of squats with a ball and overhead pressing with dumbbells. The exercises were performed progressively according to the NASM corrective exercise guidelines (Table 1) for 8 weeks, 3 days a week, under the direct supervision of the examiner.

### 3.2 The Neck and Shoulder KT Method

**Figure 3:**
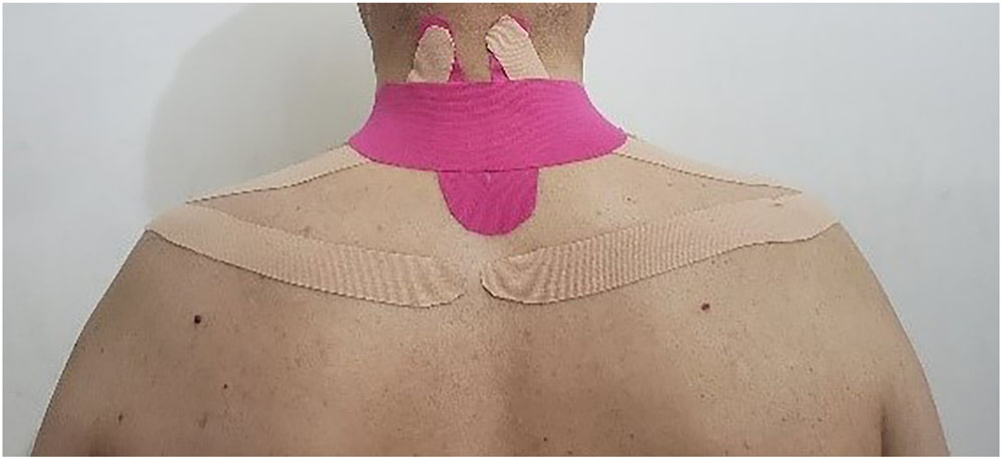
The neck and shoulder KT method.

The second part of the intervention included the use of KT for the correction of posture and prevention of poor posture during 8 week research process. Research suggests that the pressure created by KT can prevent FHP and rounded shoulders during daily activities by exerting a mechanical effect and may also boost the effectiveness of the exercise program [23]. The KT method is described below. The KT No. 1 was a U‐shaped strip, used to facilitate the contraction of the semispinalis cervical muscle; this tape was applied in the direction of muscle fibers. The base of the U‐shaped strip was in the spinous process of T2, and the arms of the strip were stretched 15%–25% below the hair growth line. The KT No. 2 was a U‐shaped strip extending across the shoulder along the upper trapezius muscle, facilitating the middle trapezius muscle. The other base of this strip was attached to the acromion appendage. The strip was applied to the upper trapezius muscle area with 10% tension. The other arm of the strip was attached above the middle trapezius muscle at 15%–25% tension. Finally, KT No. 3 was an I‐shaped strip, applied horizontally on the middle of the neck to create guiding mechanical tension for correcting FHP. The participant was asked to maintain a normal range of lordosis in the neck. Subsequently, the base of the I‐shaped strip was applied along the vertebrae (C5–C7) with 50%–75% tension at the end of the base [22]. The KT was applied two times per week, and each application was maintained for approximately 48–72 h, depending on skin tolerance and adhesion. As long as the band remained on the skin, if the strip loosened or peeled off, it would be examined and replaced by the examiner [22] (Figure 3).

### 3.3 Ethics Approval

All procedures were approved first by the Ethics Committee of Guilan University, and then by the Ethics Committee of Sport Sciences Research Institute (IR.SSRI.REC.1400.1208), whose guidelines are by the Declarations of Helsinki. All participants entered the study after completing and signing the informed consent form.

### 3.4 Statistical Analysis

The Shapiro–Wilk test was used to investigate the normal distribution of data. To examine the homogeneity of the groups, a pre‐test comparison of the two groups was carried out using an independent *t*‐test. Data were analyzed using a 2 × 2 repeated measures ANOVA followed by post hoc (Bonferroni). Post hoc tests were conducted to compare pre‐ and post‐test results within each group. Effect sizes indicate the intensity of the intervention's impact on the variables. Effect sizes partial eta squared were classified as small (0.01), moderate (0.06), and large (0.14) and effect sizes Cohen's *d*, includes small effect sizes: 0.01 to 0.19, medium: 0.20 to 0.49, and large: 0.50 and above. Findings were analyzed at a confidence level of 99%, with a statistical significance of (*p* < 0.01), and performed using SPSS software (SPSS, version 25).

## 4 Results

**The demographic characteristics of the participants.** The results of the demographic characteristics of the control and experimental groups are presented in Table 2. According to Table 1, there was no significant difference between the two groups in terms of age, height, weight, occupation, or body mass index (BMI) at the onset of the study (*p* ≥ 0.01); therefore, the two groups were homogeneous regarding these variables.

**Table 2:** The demographic characteristics of the participants, reported as mean ± standard deviations.

Measurement	group	Mean ± SD	*T*	*p*
Age (years)	CG	42.00 ± 7/71	0.97	0.92
	EG	41.73 ± 7.36		
Height (cm)	CG	1.69 ± 0.06	1.75	0.09
	EG	1.65 ± 0.06		
Weight (kg)	CG	69.60 ± 12.29	0.13	0.89
	EG	69.00 ± 11.31		
Job history (y)	CG	14.53 ± 7.79	−0.17	0.86
	EG	15.00 ± 7.17		
BMI (kg/m^2^)	CG	23.98 ± 3.06	−0.84	0.40
	EG	25.03 ± 3.64		

Abbreviations: CG, control group; EG, experimental group.

Then the results of the Shapiro–Wilks and Levene's tests confirmed that the data were normally distributed and the variances were homogeneous (*p* > 0.05) in all subjects.

As per Table 3, repeated measures ANOVA results revealed significant effects of the 8‐week selected corrective exercises with KT. The interaction effects of group × time (interaction) for all variables, including forward head, rounded shoulders, kyphosis, and scapular position at angles of 0°, 45°, and 90°, were significant with large effect sizes, indicating the specific effectiveness of the combined intervention over time compared to the control group. This interaction effects were found for the FHP (*F* = 44.54; *p* = 0.001; ES = 0.61), round shoulder (*F* = 8.34; *p* = 0.007; ES = 0.23), kyphosis (*F* = 44.79; *p* = 0.001; ES = 0.61), position 0° (*F *= 22.81; *p* = 0.001; ES = 0.44), position 45° (*F* = 16.09; *p* = 0.001; ES = 0.36), and position 90° (*F* = 13.47; *p* = 0.001; ES = 0.32). The main effect of time was significant only for the forward head angle. Additionally, significant main effects of time were found for the forward head (*F *= 7.87; *p* = 0.009; ES = 0.22). But, significant main effects of time were not found for the round shoulder (*F* = 0.43; *p* = 0.51; ES = 0.01), kyphosis (*F* = 0.40; *p* = 0.53; ES = 0.01), position 0° (*F* = 1.02; *p* = 0.31; ES = 0.03), position 45° (*F* = 0.05; *p* = 0.81; ES = 0.02), and position 90° (*F* = 1.70; *p* = 0.20; ES = 0.05). The main effect of group was significant only for the variable scapular distance at 90°. The main effect of the group was significant at the forward head (*F* = 4.94; *p* = 0.03; ES = 0.15), round shoulder (*F* = 4.79; *p* = 0.003; ES = 0.14), and position 90° (*F* = 8.60; *p* = 0.007; ES = 0.23). But, the main effect of the group was not significant at the kyphosis (*F* = 0.06; *p* = 0.79; ES = 0.02), position 0° (*F* = 0.03; *p* = 0.85; ES = 0.01), position 45° (*F* = 0.18; *p* = 0.67; ES = 0.06).

**Table 3:** Results of repeated measures ANOVA test to compare the means of variables.

Variable	Main effect of group		Main effect of time		Interaction effect (time × group)	
	*p* value	ES	*p* value	ES	*p* value	ES
FHP (°)	0.03	Medium (0.15)	0.009*	Medium (0.22)	0.001*	Large (0.61)
RS (°)	0.03	Small (0.14)	0.51	Small (0.01)	0.007*	Medium (0.23)
Kyphosis angle (°)	0.79	Small (0.02)	0.53	Small (0.01)	0.001*	Large (0.61)
Scapular distance at 0° (cm)	0.85	Small (0.01)	0.31	Small (0.03)	0.001*	Medium (0.44)
Scapular distance at 45° (cm)	0.67	Small (0.06)	0.81	Small (0.02)	0.001*	Medium (0.36)
Scapular distance at 90° (cm)	0.007*	Medium (0.23)	0.20	Small (0.05)	0.001*	Medium (0.32)

Abbreviations: CG = control group, EG = experimental group, ES = effect size, FHP = forward head posture, RS = round shoulder, SD = standard deviation.

* Significance at the *p* < 0.01 level.

As per Table 4, the greatest effect of the intervention was related to the FHP in the experimental group with a large effect size (0.61). Other variables also showed mainly moderate effects. In the control group, most of the changes were small or in a negative direction. These results indicate the favorable effectiveness of the combined intervention of corrective exercises and Kinesio taping in improving physical posture indicators. The results of the pre‐ and post‐test post hoc of the experimental group, except for the round shoulder variable, were significant for all variables with a large effect size. Post hoc tests showed significant differences in the forward head (*F* = 44.94; *p* = 0.001; ES = 0.61), round shoulder (*F* = 6.30; *p* = 0.01; ES = 0.18), kyphosis (*F* = 18.36; *p* = 0.001; ES = 0.39), Position 0° (*F* = 17.67; *p* = 0.001; ES = 0.37), position 45° (*F* = 9.02; *p* = 0.006; ES = 0.24), and position 90° (*F* = 12.37; *p* = 0.002; ES = 0.30) in the experimental group compared to the control group. However, there was significant difference between the pre‐test and the Posttest in the forward head (*F* = 7.47; *p* = 0.01; ES = 0.21), kyphosis (*F* = 28.82; *p* = 0.001; ES = 0.48), position 0° (*F* = 7.07; *p* = 0.01; ES = 0.20), and position 45° (*F* = 7.09; *p* = 0.01; ES = 0.20) in the control group. However, the presence of a significant difference in the control group was due to the weaker records in posttest (FHP, Kyphosis, Scapula 0°,45°) of the subjects, which inversely made the results significant in this group (Table 4).

**Table 4:** Intra‐group comparison of pre‐ and post‐test results with post hoc testing.

Variables	Group	Pre‐test mean ± SD	Posttest mean ± SD	*F*	*p* value	ES	Effect size interpretation
FHP (degree)	CG	55.13 ± 4.05	57.20 ± 4.16	7.47	0.01	0.21	Small to moderate (worsening)
	EG	55.46 ± 3.97	50.40 ± 4.73	44.94	0.001*	0.61	Large (improvement)
RS (degree)	CG	10.44 ± 2.15	10.70 ± 2.23	2.47	0.12	0.08	Very small
	EG	9.28 ± 1.63	8.86 ± 1.52	6.30	0.01	0.18	Small (improvement)
Kyphosis (degree)	CG	54.04 ± 7.22	59.68 ± 6.54	28.82	0.001*	0.48	Moderate to large (worsening)
	EG	58.47 ± 8.89	53.81 ± 8.80	18.36	0.001*	0.39	Moderate (improvement)
Scapula 0° (cm)	CG	8.27 ± 0.74	8.61 ± 0.55	7.07	0.01	0.20	Small to moderate (mild worsening)
	EG	8.64 ± 0.91	8.12 ± 1.09	16.76	0.001*	0.37	Moderate (improvement)
Scapula 45° (cm)	CG	9.06 ± 0.85	9.50 ± 0.99	7.09	0.01	0.20	Small to moderate (worsening)
	EG	9.39 ± 1.01	8.89 ± 0.98	9.02	0.006*	0.24	Small to moderate (improvement)
Scapula 90° (cm)	CG	10.58 ± 1.01	10.87 ± 0.90	2.79	0.10	0.09	Very small
	EG	10.05 ± 1.08	9.94 ± 0.88	12.37	0.002*	0.30	Moderate (improvement)

Abbreviations: CG = control group, EG = experimental group, ES = effect size, FHP = forward head posture, RS = round shoulder; SD = standard deviation.

* Significance at the *p* < 0.01 level.

## 5 Discussion

This study examined the effects of an 8‐week program of selected corrective exercises combined with the KT on postural alignment in dentists with FHP. The results demonstrated significant improvements in forward head angle, rounded shoulders, kyphosis angle, and scapular positioning in the experimental group from pre‐ to post‐intervention. Furthermore, post‐test comparisons between the experimental and control groups revealed significant differences across all measured variables, with the experimental group showing superior outcomes. These findings suggest that the combined intervention was effective in improving postural alignment in this population. Moreover, the analysis of effect sizes revealed significant changes in all the mentioned variables, among which the forward head angle and kyphosis showed the greatest changes. An explanation for the observed local effect may be the long‐term efficacy of KT in correcting posture and preventing poor posture in daily activities during the research, since the combination of KT with corrective exercises could enhance and accelerate the efficacy of corrective exercises, although further relevant research is needed. It was also observed that the change in the rounded shoulder angle after the intervention was more limited than the improvement in forward head posture and kyphosis angle. This finding may be related to several factors. One possible reason is the chronic tightness of the pectoralis major and minor muscles in individuals who remain in a shortened position due to long‐term occupational postures. Therefore, the 8‐week intervention period may not have been sufficient to create effective and lasting stretching in these muscles, and longer intervention periods and specific stretching exercises with higher intensity and repetition are probably needed.

On the other hand, in the present protocol, the main part of the passive intervention consisted of the use of taping tape, which mainly focused on the neck and back area. Therefore, it can be concluded that adding more specialized stretching exercises for the pectoralis muscles with a longer duration may provide better results in correcting rounded shoulder posture. On the other hand, the results of this study showed that the control group had significant changes in forward head posture and scapular position of 0° and 45° in the direction of poor posture. These results confirm the progression of postural disorders, especially in the cervical and thoracic spine areas, following the maintenance of long‐term bent posture during the daily work of dentists.

In agreement, in the Russo et al. study on rugby players using an acute combined exercise and taping most of investigated variables were improved compared to the separate exercise and taping groups [48]. Also, in the Gandolfi study, a comprehensive yoga protocol was designed for dentists, consisting of more than 60 selected asanas suitable for the dental office environment. The protocol focused on the upper body areas, including the neck, upper back, shoulders, and chest. Areas that are most affected by occupational musculoskeletal disorders in dentists. Designed to release muscles, oxygenate, and reduce joint and muscle strain. The designed protocol is a practical and applicable guide for the prevention and treatment of occupational musculoskeletal disorders in dentists and other professionals who are exposed to prolonged positions and poor postures [27, 49].

It is well‐known that FHP is one of the most common postural abnormalities among dentists. Commonly, a set of neuromuscular disorders occurs as a chain reaction of the joints, muscles, and nerves, which may affect posture and cause muscle imbalance in the upper quarter of the body [50, 51]; therefore, researchers need to design exercises according to this chain reaction. In this regard, in a study, the use of KT on cervical region improved the active range of motion of muscles and alleviated the cervical spine pain [52]. Considering the prolonged spinal misalignment of dentists during work, the use of KT as a supportive method may increase the effectiveness of corrective exercises for the neck and shoulder girdle and help correct their posture. Also, chin tucks, with and without theraband, were one of the main exercises of the training program. Based on previous research, the chin tuck exercise alone does not have durable effects; accordingly, we combined this exercise with other corrective exercises. As mentioned earlier, we tried to design the exercise program based on the National American Academy of Sports Medicine (NASM). In this method, which involves four techniques of inhibition, lengthening, activation, and integration, it is recommended to first perform inhibition exercises, followed by stretching exercises on the muscle rather than simply stretching the shortened or tightened muscle [32]. Overall, a proper corrective exercise program, aimed at correcting FHP‐related deformities (kyphosis, rounded shoulder, and scapular position), can correct the cervical spine of individuals. A comprehensive and appropriate corrective program for FHP can lead to the simultaneous correction of related deformities. Besides, the present results about the effects of corrective exercises on shoulder dyskinesia are in line with the findings of studies by Yoosefi et al., Abdullahzadeh et al. and Pavan Kumar et al. [33, 53, 54].

In addition to the effects of corrective exercises, the KT method had several advantages, such as improvement of blood and lymph circulation, pain alleviation through neurological effects, joint positioning, facilitation and inhibition of muscle activities, prevention of damage, modification of proprioception, and improvement of muscle function and fascia [17, 48]. Also, the inhibitory effect of the KT to prevent the tendency to put the head and spine forward can help maintain the correct alignment of the body throughout the day while performing daily activities [55]. Considering the advantages of KT in improving muscular tension, alleviating trigger points and extending the range of motion, it can be applied by itself or mixed with other therapeutic modalities [56]. Research continues to design and use targeted and effective active and inactive interventions [13, 14, 31, 57, 58], as Öztürk et al. on the short‐term and medium‐term effects of the KT technique on the trapezius muscle in people with myofascial pain syndrome. The use of KT in people with myofascial pain syndrome resulted in statistically significant improvements in pain reduction and increased strength of the upper trapezius muscle [59].

Based on the findings of this study, the combined use of kinesiology taping and corrective exercises appears to be a practical and effective intervention for individuals who maintain prolonged poor posture, such as dentists and computer users. The observed improvements were achieved through the simultaneous application of both corrective exercises and KT. However, because both interventions were applied concurrently, it was not possible to determine the individual contribution of each component. Therefore, it cannot be concluded that the combined approach is superior to corrective exercises alone. Given the significant effectiveness of the combined intervention of comprehensive corrective exercises with KT in improving postural indices, it is recommended that this protocol be used as part of prevention and treatment programs in occupational settings with a high risk of musculoskeletal disorders, especially for dentists, hairdressers, and other professionals in static occupations such as surgeons. For greater effectiveness, regular implementation of corrective exercises under the supervision of corrective movement specialists and physiotherapists is recommended. Also, future studies can investigate the durability of the intervention effects using placebo‐controlled designs and long‐term follow‐up.

## 6 Limitation

The limitations of our study include the use of only a combination of interventions in separate groups. Another limitation of this study was the lack of blinding of participants and assessors. Given the nature of the intervention, which required the implementation of corrective exercises and typing under the close supervision of the researcher, blinding was not possible. This may have led to some performance or assessment bias. However, to reduce the impact of this limitation, standardized protocols and objective measurement tools were used. One of the limitations of this study is the absence of a placebo or sham taping control group, which restricts the ability to distinguish between the physiological effects of kinesio taping and placebo responses. Given the visible and tangible nature of the taping, some outcomes may have been influenced by participants' subjective expectations. Therefore, it is recommended that future studies employ placebo‐controlled designs to better differentiate the true effects of the intervention from placebo effects. Also, there was no follow‐up of results to assess the sustainability of effects in the months following the intervention.

## 7 Conclusion

The findings of this study emphasize the effectiveness of a combined intervention including comprehensive posture correction exercises focusing on releasing, strengthening, and integrating the muscles involved in the postural alignment of the body, along with KT. This protocol not only improved postural alignment, such as head forward, rounded shoulders, and kyphosis, but also promoted the maintenance of proper posture and facilitated the neuromuscular retraining process by providing continuous proprioceptive feedback through taping. From a practical perspective, these findings can serve as a basis for designing targeted preventive and therapeutic programs for dentists and other professionals who are exposed to long‐term static positions and are at risk of postural abnormalities. Utilizing such combined protocols in high‐risk work environments can play an important role in reducing musculoskeletal complications and improving professional performance.

## Author Contributions

**Zahra Fadaei Forghan:** methodology, formal analysis, conceptualization, data curation, software, validation, writing – original draft, and project administration. **Parisa Sedaghati:** supervision, resources, formal analysis, investigation, writing – original draft, writing – review and editing, visualization, software, validation, conceptualization, methodology, data curation, and project administration.

## Ethics Statement

The authors confirm that all phases of the research were done under relevant guidelines and regulations. Whose guidelines are by the Declarations of Helsinki. The current research was approved by the Sport Sciences Research Institute (code: IR.SSRI.REC.2021.1208). This study was taken from the MSc. thesis on corrective exercise and sport injury at the University of Guilan (code: 17186; dated: 13.02.2023).

## Consent

All participants were conducted with the adequate understanding and contributed to this study voluntarily after completing and signing the informed consent form.

## Conflicts of Interest

The authors declare no conflicts of interest.

## Transparency Statement

The lead author, P. Sedaghati, affirms that this manuscript is an honest, accurate, and transparent account of the study being reported; that no important aspects of the study have been omitted; and that any discrepancies from the study as planned (and, if relevant, registered) have been explained.

## Data Availability

Data sharing is not applicable to this article as no data sets were generated or analyzed during the current study.
